# 6,7-Di­chloro-2-methyl-3-phenyl­quinoxaline

**DOI:** 10.1107/S2414314626008928

**Published:** 2026-09-03

**Authors:** Myron W. Jones, Katelyn M. Aldridge, Guy Crundwell

**Affiliations:** a Department of Chemistry Southern Illinois University Edwardsville,Edwardsville IL 62026-1652 USA; b Department of Chemistry & Biochemistry Central Connecticut State University, 1619 Stanley Street New Britain CT 06053 USA; Purdue University, USA

**Keywords:** crystal structure, quinoxaline

## Abstract

The title compound, C_15_H_10_Cl_2_N_2_, was synthesized using the acid catalyst NH_4_HF_2_ in a water/methanol solution. The dihedral angle between the phenyl ring and the quinoxaline unit is 39.80 (5)°. There is an inter­molecular hydrogen-bonding-like inter­action with a neighboring chlorine atom at a distance of 2.76 Å. The extended structure features offset-stacked quinoxaline units along the [100] direction.

## Abstract 2

The title compound was synthesized using the acid catalyst NH_4_HF_2_ in a water/methanol solution. The extended structure features offset stacked quinoxalines along the [100] direction.

## Graphical abstract

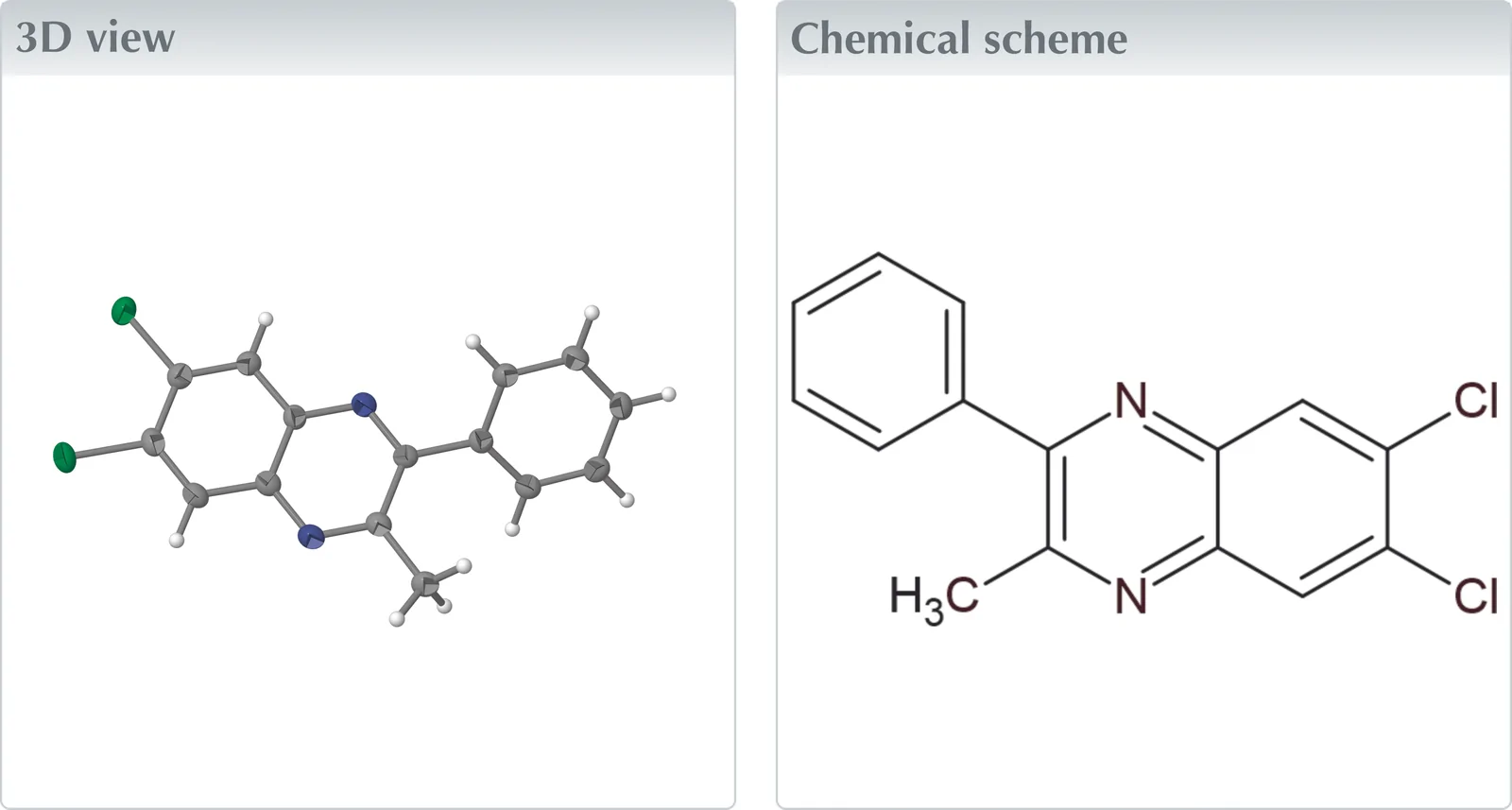

## Structure description

In general, quinoxalines are of inter­est as potential anti­bacterial (Parhi *et al.*, 2013 ▸), anti­microbial (Singh *et al.*, 2010 ▸), and anti­fungal medicines (Tang *et al.*, 2022 ▸) and therefore exploring their synthesis and structures are of inter­est. The title compound was synthesized via a condensation reaction of a di­amine and diketone using a water/methanol solution containing the NH_4_HF_2_ catalyst (Lassagne *et al.*, 2015 ▸).

**Figure 1:**
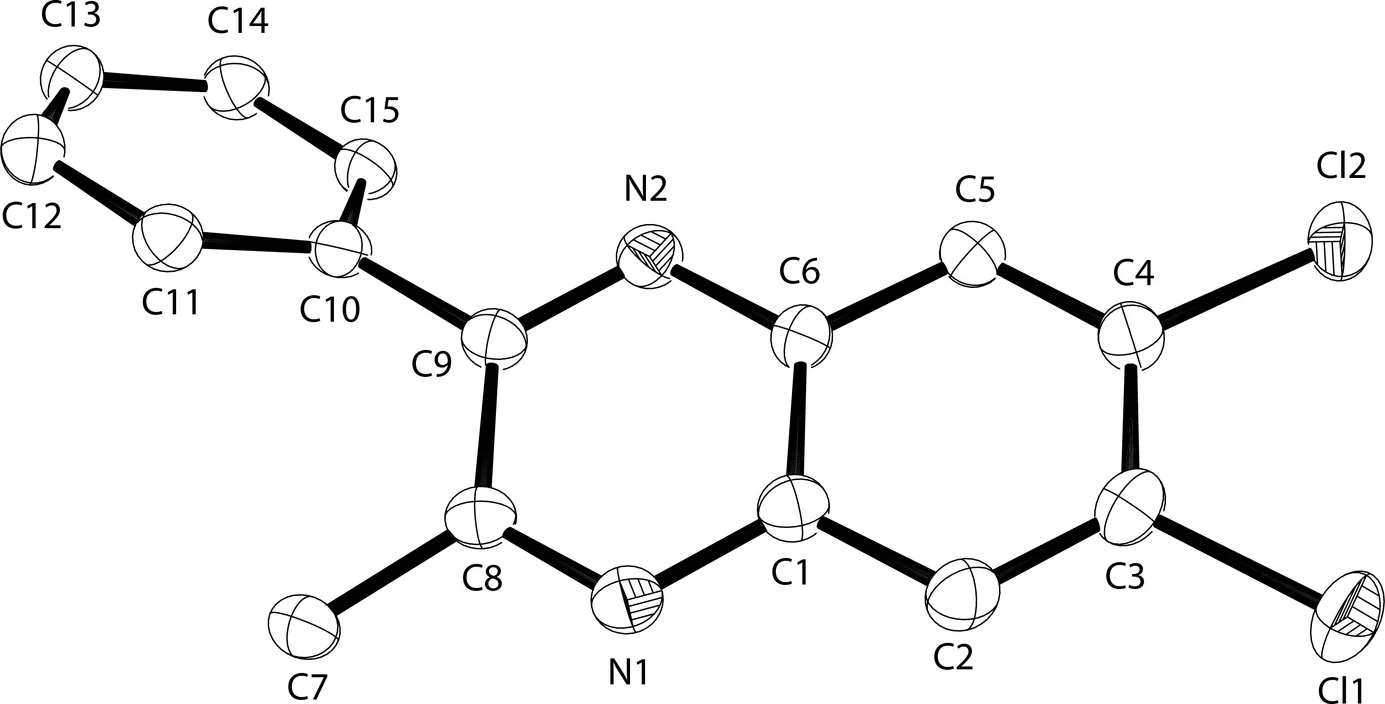
A view of the title molecule. Hydrogen atoms are omitted and displacement ellipsoids are drawn at the 50% probability level.

**Figure 2:**
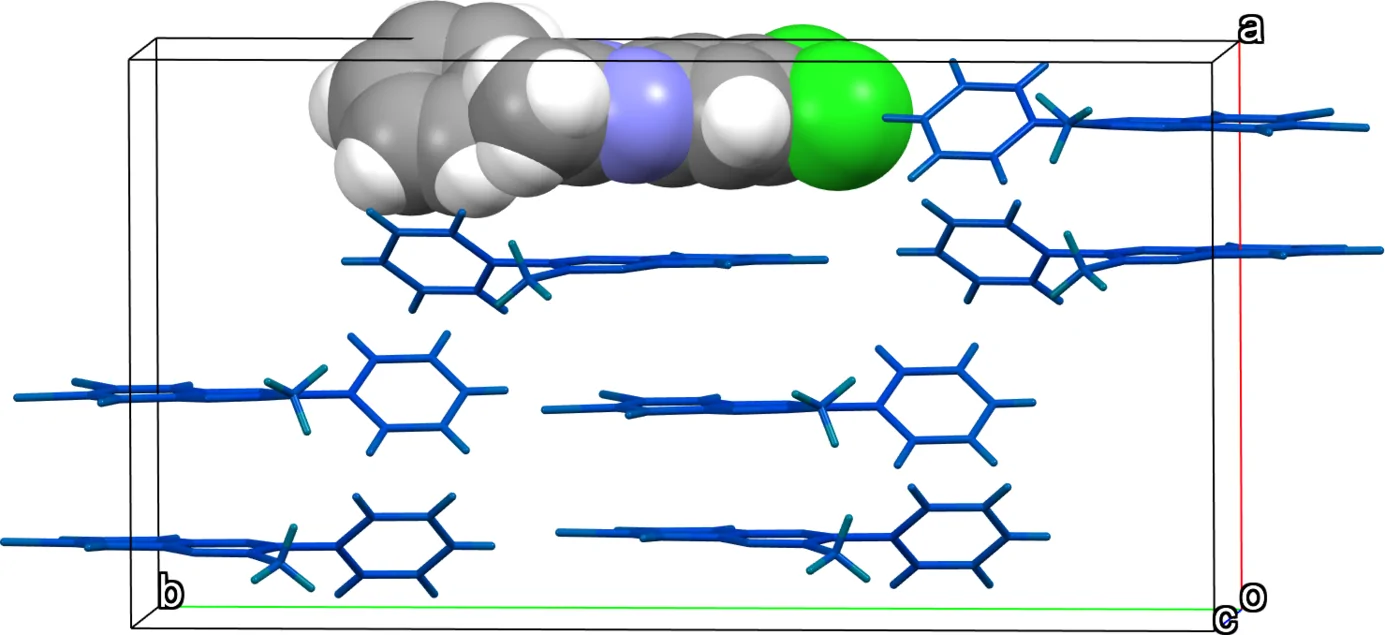
A view of the unit-cell packing along the [100] direction.

The mol­ecular structure is shown in Fig. 1 ▸. The quinoxaline ring system is essentially planar. The r.m.s. deviation of the di­chloro­quinoxaline unit (using atoms C1–C9, N1, N2, Cl1, and Cl2) is only 0.0604 Å. The quinoxaline moiety makes an angle of 39.80 (5)° with respect to the phenyl ring. There is a hydrogen-bonding-like inter­action between a carbon hydrogen and a neighboring chlorine atom (Table 1 ▸). Mol­ecules stack along the [100] direction of the unit cell (Fig. 2 ▸). Analysis of ring inter­actions using *PLATON* (Spek, 2009 ▸) indicats that the quinoxaline ring system (C1–C6,C8,C9,N1,N2) exhibits π⋯π stacking with inter­planar spacings of 3.5039 (7) Å [for neighbor at (1 − *x*, −*y*, *z*)], 3.4022 (7) Å [for neighbor at (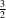 − *x*, *y*, −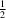 + *z*)], and 3.3845 (7) Å [for neighbor at (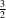 − *x*, *y*, 1/2 *+* z)] with slippages of 1.515, 3.582, and 3.565Å, respectively..

## Synthesis and crystallization

In a 50 mL Erlenmeyer flask, 1.49 g of 1-phenyl-1,2-propane­dione (10.0 mmol) were added to 2 mL of H_2_O and 20 mL of a 2.5 x 10^−3^*M* methanol solution of NH_4_HF_2_ (Lassagne *et al.*, 2015 ▸). To this mixture 1.78 g of 4,5-di­chloro-1,2-phenyl­enedi­amine (10.0 mmol) were added. The solution was stirred for 24 h during which the product precipitated as a pale light-tan product. 2.65 g of crude product were isolated and recrysallized from methanol solution yielding 1.92 g of purified product (66%). Crystals for the diffraction study were isolated by the slow evaporation of 50/50 hexa­ne/ethyl acetate solutions. M.p: 426 K; ^1^H NMR (CDCl_3_, 300 MHz): δ = 2.78 (*s*, 3H), 7.55 (*m*, 3H), 7.64 (*m*, 2H), 8.17 (*s*, 1H), 8.22 (*s*, 1H); ^13^C NMR (CDCl_3_, 300 MHz): δ = 24.51, 128.85, 128.91, 129.16, 129.43, 129.84, 133.65, 134.12, 138.31, 139.77, 139.86, 153.98, 155.89.

## Refinement

Crystal data, data collection and structure refinement details are summarized in Table 2 ▸.

## Supplementary Material

Crystal structure: contains datablock(s) I. DOI: 10.1107/S2414314626008928/zl4103sup1.cif

Structure factors: contains datablock(s) I. DOI: 10.1107/S2414314626008928/zl4103Isup2.hkl

Supporting information file. DOI: 10.1107/S2414314626008928/zl4103Isup3.cml

CCDC reference: 2583897

Additional supporting information:  crystallographic information; 3D view; checkCIF report

## Figures and Tables

**Table 1:** Hydrogen-bond geometry (Å, °)

*D*—H⋯*A*	*D*—H	H⋯*A*	*D*⋯*A*	*D*—H⋯*A*
C2—H2⋯Cl2^i^	0.95	2.76	3.704 (3)	175

Symmetry code: (i) 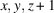.

**Table 2:** Experimental details

Crystal data	
Chemical formula	C_15_H_10_Cl_2_N_2_
*M*_r_	289.15
Crystal system, space group	Orthorhombic, *Aea*2
Temperature (K)	150
*a*, *b*, *c* (Å)	13.7569 (4), 26.2508 (8), 7.1470 (2)
*V* (Å^3^)	2580.99 (13)
*Z*	8
Radiation type	Cu *K*α
μ (mm^−1^)	4.40
Crystal size (mm)	0.21 × 0.11 × 0.06

Data collection	
Diffractometer	Bruker AXS D8 Quest
Absorption correction	Multi-scan (*SADABS*; Krause *et al.*, 2015 ▸)
*T*_min_, *T*_max_	0.500, 0.754
No. of measured, independent and observed [*I* > 2σ(*I*)] reflections	35150, 2788, 2646
*R*_int_	0.064
(sin θ/λ)_max_ (Å^−1^)	0.640

Refinement	
*R*[*F*^2^ > 2σ(*F*^2^)], *wR*(*F*^2^), *S*	0.029, 0.078, 1.11
No. of reflections	2788
No. of parameters	173
No. of restraints	1
H-atom treatment	H-atom parameters constrained
Δρ_max_, Δρ_min_ (e Å^−3^)	0.23, −0.28
Absolute structure	Flack *x* determined using 1146 quotients [(*I*^+^)−(*I*^−^)]/[(*I*^+^)+(*I*^−^)] (Parsons *et al.*, 2013 ▸)
Absolute structure parameter	0.004 (7)

Computer programs: *APEX6* and *SAINT* (Bruker, 2026 ▸), *SHELXT* (Sheldrick, 2015*a* ▸), *SHELXL2025/1* (Sheldrick, 2015*b* ▸), *OLEX2* (Dolomanov *et al.*, 2009 ▸), *ORTEP-3 for Windows* (Farrugia, 2012 ▸) and *Mercury* (Macrae *et al.*, 2020 ▸).

## full crystallographic data

### 6,7-Dichloro-2-methyl-3-phenylquinoxaline Crystal data

**Table:**

C_15_H_10_Cl_2_N_2_	*D*_x_ = 1.488 Mg m^−^^3^
*M**_r_* = 289.15	Melting point: 426 K
Orthorhombic, *A**e**a*2	Cu *K*α radiation, λ = 1.54178 Å
*a* = 13.7569 (4) Å	Cell parameters from 9889 reflections
*b* = 26.2508 (8) Å	θ = 3.4–80.1°
*c* = 7.1470 (2) Å	µ = 4.40 mm^−^^1^
*V* = 2580.99 (13) Å^3^	*T* = 150 K
*Z* = 8	Plate, clear colourless
*F*(000) = 1184	0.21 × 0.11 × 0.06 mm

### 6,7-Dichloro-2-methyl-3-phenylquinoxaline Data collection

**Table:**

Bruker AXS D8 Quest diffractometer	2788 independent reflections
Radiation source: I-mu-S 3.0 microsource X-ray tube	2646 reflections with *I* > 2σ(*I*)
HELIOS multilayer Montel optics monochromator	*R*_int_ = 0.064
Detector resolution: 7.4074 pixels mm^-1^	θ_max_ = 80.6°, θ_min_ = 3.4°
ω and phi scans	*h* = −17→17
Absorption correction: multi-scan (SADABS; Krause *et al.*, 2015)	*k* = −33→33
*T*_min_ = 0.500, *T*_max_ = 0.754	*l* = −8→9
35150 measured reflections	

### 6,7-Dichloro-2-methyl-3-phenylquinoxaline Refinement

**Table:**

Refinement on *F*^2^	Secondary atom site location: difference Fourier map
Least-squares matrix: full	Hydrogen site location: inferred from neighbouring sites
*R*[*F*^2^ > 2σ(*F*^2^)] = 0.029	H-atom parameters constrained
*wR*(*F*^2^) = 0.078	*w* = 1/[σ^2^(*F*_o_^2^) + (0.0438*P*)^2^ + 0.4866*P*] where *P* = (*F*_o_^2^ + 2*F*_c_^2^)/3
*S* = 1.11	(Δ/σ)_max_ = 0.001
2788 reflections	Δρ_max_ = 0.23 e Å^−^^3^
173 parameters	Δρ_min_ = −0.28 e Å^−^^3^
1 restraint	Absolute structure: Flack *x* determined using 1146 quotients [(*I*^+^)-(*I*^-^)]/[(*I*^+^)+(*I*^-^)] (Parsons *et al.*, 2013)
Primary atom site location: dual	Absolute structure parameter: 0.004 (7)

### 6,7-Dichloro-2-methyl-3-phenylquinoxaline Special details

**Table:**

Geometry. All esds (except the esd in the dihedral angle between two l.s. planes) are estimated using the full covariance matrix. The cell esds are taken into account individually in the estimation of esds in distances, angles and torsion angles; correlations between esds in cell parameters are only used when they are defined by crystal symmetry. An approximate (isotropic) treatment of cell esds is used for estimating esds involving l.s. planes.
Refinement. Hydrogen atoms on sp^2^ and sp^3^ carbons were placed at calculated positions with a C-H distance of 0.95 Å and 0.98 Å and were included in the refinement in riding motion approximation with U_iso_ = 1.2U_eq_ or 1.5U_eq_ of the carrier atom, respectively.

### 6,7-Dichloro-2-methyl-3-phenylquinoxaline Fractional atomic coordinates and isotropic or equivalent isotropic displacement parameters (Å^2^)

**Table:**

	*x*	*y*	*z*	*U*_iso_*/*U*_eq_	
C1	0.37186 (15)	0.47901 (10)	0.6000 (4)	0.0262 (5)	
C3	0.37034 (16)	0.56404 (10)	0.4771 (4)	0.0284 (5)	
C10	0.37433 (14)	0.32221 (9)	0.4945 (4)	0.0242 (5)	
C15	0.42966 (17)	0.30390 (9)	0.3437 (3)	0.0258 (5)	
H15	0.466810	0.327048	0.270755	0.031*	
N1	0.37599 (15)	0.44750 (9)	0.7518 (3)	0.0280 (5)	
Cl1	0.36731 (4)	0.62940 (2)	0.51359 (11)	0.03628 (18)	
C13	0.37601 (17)	0.21837 (10)	0.4054 (4)	0.0303 (5)	
H13	0.377408	0.183049	0.376637	0.036*	
C6	0.37267 (16)	0.45912 (9)	0.4161 (4)	0.0242 (5)	
C12	0.31946 (17)	0.23595 (9)	0.5524 (3)	0.0306 (5)	
H12	0.281249	0.212716	0.622943	0.037*	
Cl2	0.37977 (5)	0.58627 (2)	0.10644 (10)	0.03599 (18)	
C4	0.37379 (16)	0.54433 (10)	0.2935 (4)	0.0275 (5)	
C5	0.37397 (16)	0.49310 (10)	0.2613 (4)	0.0267 (5)	
H5	0.374956	0.480307	0.136961	0.032*	
C8	0.37819 (16)	0.39822 (10)	0.7220 (4)	0.0250 (5)	
C14	0.43071 (17)	0.25251 (10)	0.3002 (4)	0.0291 (5)	
H14	0.468808	0.240553	0.198416	0.035*	
N2	0.37302 (13)	0.40786 (8)	0.3852 (3)	0.0259 (5)	
C2	0.36965 (16)	0.53235 (11)	0.6280 (4)	0.0294 (5)	
H2	0.367700	0.545967	0.751059	0.035*	
C9	0.37394 (14)	0.37773 (10)	0.5335 (4)	0.0232 (5)	
C11	0.31853 (16)	0.28751 (8)	0.5968 (4)	0.0276 (4)	
H11	0.279563	0.299277	0.697663	0.033*	
C7	0.38880 (19)	0.36530 (10)	0.8927 (4)	0.0299 (5)	
H7A	0.325163	0.351482	0.927622	0.045*	
H7B	0.414578	0.385727	0.996143	0.045*	
H7C	0.433567	0.337216	0.865550	0.045*	

### 6,7-Dichloro-2-methyl-3-phenylquinoxaline Atomic displacement parameters (Å^2^)

**Table:**

	*U*^11^	*U*^22^	*U*^33^	*U*^12^	*U*^13^	*U*^23^
C1	0.0260 (11)	0.0266 (13)	0.0260 (11)	0.0011 (8)	0.0006 (10)	−0.0021 (11)
C3	0.0277 (11)	0.0226 (13)	0.0350 (14)	−0.0003 (8)	0.0032 (9)	−0.0036 (10)
C10	0.0257 (10)	0.0242 (11)	0.0228 (12)	0.0017 (7)	−0.0034 (8)	−0.0012 (10)
C15	0.0287 (10)	0.0266 (11)	0.0221 (10)	0.0002 (9)	−0.0007 (8)	0.0006 (8)
N1	0.0320 (10)	0.0285 (11)	0.0235 (10)	0.0005 (8)	0.0006 (7)	−0.0029 (9)
Cl1	0.0454 (3)	0.0214 (3)	0.0421 (4)	−0.0014 (2)	0.0073 (3)	−0.0051 (2)
C13	0.0405 (13)	0.0215 (13)	0.0289 (13)	0.0001 (9)	−0.0036 (11)	−0.0014 (10)
C6	0.0256 (11)	0.0218 (12)	0.0252 (11)	0.0012 (8)	−0.0003 (8)	−0.0007 (10)
C12	0.0354 (12)	0.0287 (12)	0.0278 (13)	−0.0049 (9)	0.0008 (8)	0.0018 (8)
Cl2	0.0506 (3)	0.0257 (3)	0.0316 (3)	−0.0025 (2)	−0.0014 (3)	0.0041 (2)
C4	0.0281 (11)	0.0266 (13)	0.0278 (13)	−0.0002 (9)	−0.0006 (9)	0.0014 (10)
C5	0.0303 (12)	0.0256 (12)	0.0241 (13)	−0.0006 (9)	−0.0005 (8)	0.0000 (10)
C8	0.0249 (11)	0.0268 (12)	0.0234 (12)	0.0001 (8)	0.0003 (8)	−0.0026 (10)
C14	0.0332 (11)	0.0294 (11)	0.0246 (10)	0.0028 (10)	0.0008 (9)	−0.0028 (9)
N2	0.0281 (10)	0.0256 (12)	0.0240 (11)	0.0018 (7)	−0.0002 (8)	−0.0016 (8)
C2	0.0346 (12)	0.0270 (13)	0.0267 (14)	0.0006 (8)	0.0027 (9)	−0.0056 (10)
C9	0.0225 (9)	0.0252 (12)	0.0220 (11)	0.0005 (7)	0.0005 (8)	0.0001 (9)
C11	0.0292 (10)	0.0281 (11)	0.0257 (10)	0.0000 (8)	0.0019 (9)	−0.0018 (9)
C7	0.0374 (12)	0.0296 (14)	0.0226 (12)	0.0005 (10)	−0.0029 (10)	−0.0008 (10)

### 6,7-Dichloro-2-methyl-3-phenylquinoxaline Geometric parameters (Å, º)

**Table:**

C1—N1	1.365 (4)	C6—N2	1.364 (3)
C1—C6	1.415 (4)	C12—H12	0.9500
C1—C2	1.415 (4)	C12—C11	1.390 (3)
C3—Cl1	1.736 (3)	Cl2—C4	1.734 (3)
C3—C4	1.411 (4)	C4—C5	1.364 (4)
C3—C2	1.362 (4)	C5—H5	0.9500
C10—C15	1.404 (3)	C8—C9	1.451 (4)
C10—C9	1.484 (3)	C8—C7	1.502 (4)
C10—C11	1.397 (3)	C14—H14	0.9500
C15—H15	0.9500	N2—C9	1.323 (4)
C15—C14	1.385 (3)	C2—H2	0.9500
N1—C8	1.311 (3)	C11—H11	0.9500
C13—H13	0.9500	C7—H7A	0.9800
C13—C12	1.386 (4)	C7—H7B	0.9800
C13—C14	1.391 (4)	C7—H7C	0.9800
C6—C5	1.421 (3)		
			
N1—C1—C6	121.0 (2)	C6—C5—H5	120.4
N1—C1—C2	119.2 (3)	C4—C5—C6	119.2 (2)
C6—C1—C2	119.8 (3)	C4—C5—H5	120.4
C4—C3—Cl1	120.2 (2)	N1—C8—C9	121.0 (2)
C2—C3—Cl1	119.0 (2)	N1—C8—C7	116.0 (2)
C2—C3—C4	120.8 (2)	C9—C8—C7	123.0 (2)
C15—C10—C9	118.8 (2)	C15—C14—C13	120.0 (2)
C11—C10—C15	118.4 (2)	C15—C14—H14	120.0
C11—C10—C9	122.7 (2)	C13—C14—H14	120.0
C10—C15—H15	119.6	C9—N2—C6	117.4 (2)
C14—C15—C10	120.8 (2)	C1—C2—H2	120.2
C14—C15—H15	119.6	C3—C2—C1	119.5 (2)
C8—N1—C1	118.0 (2)	C3—C2—H2	120.2
C12—C13—H13	120.0	C8—C9—C10	122.6 (2)
C12—C13—C14	119.9 (2)	N2—C9—C10	115.9 (2)
C14—C13—H13	120.0	N2—C9—C8	121.5 (2)
C1—C6—C5	119.5 (2)	C10—C11—H11	119.7
N2—C6—C1	121.0 (2)	C12—C11—C10	120.7 (2)
N2—C6—C5	119.6 (2)	C12—C11—H11	119.7
C13—C12—H12	119.9	C8—C7—H7A	109.5
C13—C12—C11	120.1 (2)	C8—C7—H7B	109.5
C11—C12—H12	119.9	C8—C7—H7C	109.5
C3—C4—Cl2	119.1 (2)	H7A—C7—H7B	109.5
C5—C4—C3	121.2 (2)	H7A—C7—H7C	109.5
C5—C4—Cl2	119.7 (2)	H7B—C7—H7C	109.5
			
C1—N1—C8—C9	−1.5 (3)	C6—N2—C9—C10	−179.98 (18)
C1—N1—C8—C7	176.4 (2)	C6—N2—C9—C8	−2.3 (3)
C1—C6—C5—C4	−0.4 (3)	C12—C13—C14—C15	0.8 (4)
C1—C6—N2—C9	−0.9 (3)	Cl2—C4—C5—C6	177.70 (17)
C3—C4—C5—C6	−1.3 (3)	C4—C3—C2—C1	−0.4 (4)
C10—C15—C14—C13	0.5 (3)	C5—C6—N2—C9	178.54 (19)
C15—C10—C9—C8	−139.0 (2)	C14—C13—C12—C11	−1.1 (4)
C15—C10—C9—N2	38.6 (3)	N2—C6—C5—C4	−179.9 (2)
C15—C10—C11—C12	1.3 (3)	C2—C1—N1—C8	−179.85 (19)
N1—C1—C6—C5	−176.4 (2)	C2—C1—C6—C5	1.7 (3)
N1—C1—C6—N2	3.1 (3)	C2—C1—C6—N2	−178.80 (19)
N1—C1—C2—C3	176.8 (2)	C2—C3—C4—Cl2	−177.28 (18)
N1—C8—C9—C10	−178.79 (18)	C2—C3—C4—C5	1.7 (4)
N1—C8—C9—N2	3.7 (3)	C9—C10—C15—C14	−178.8 (2)
Cl1—C3—C4—Cl2	2.5 (3)	C9—C10—C11—C12	178.5 (2)
Cl1—C3—C4—C5	−178.48 (17)	C11—C10—C15—C14	−1.5 (3)
Cl1—C3—C2—C1	179.85 (16)	C11—C10—C9—C8	43.8 (3)
C13—C12—C11—C10	0.0 (4)	C11—C10—C9—N2	−138.6 (2)
C6—C1—N1—C8	−1.7 (3)	C7—C8—C9—C10	3.4 (3)
C6—C1—C2—C3	−1.3 (3)	C7—C8—C9—N2	−174.1 (2)

### 6,7-Dichloro-2-methyl-3-phenylquinoxaline Hydrogen-bond geometry (Å, º)

**Table:**

*D*—H···*A*	*D*—H	H···*A*	*D*···*A*	*D*—H···*A*
C2—H2···Cl2^i^	0.95	2.76	3.704 (3)	175

Symmetry code: (i) *x*, *y*, *z*+1.
